# Poly[[diaqua­(μ_4_-1*H*-benzimidazole-5,6-dicarboxyl­ato)strontium] monohydrate]

**DOI:** 10.1107/S1600536809048284

**Published:** 2009-11-21

**Authors:** Wen-Dong Song, Hao Wang, Juan-Hua Liu, Xiao-Tian Ma, Seik Weng Ng

**Affiliations:** a College of Science, Guangdong Ocean University, Zhanjiang 524088, People’s Republic of China; b College of Food Science and Technology, Guangdong Ocean University, Zhanjiang 524088, People’s Republic of China; c Department of Chemistry, University of Malaya, 50603 Kuala Lumpur, Malaysia

## Abstract

Each of the carboxyl­ate –CO_2_ fragments of the dianion ligand in the title compound, {[Sr(C_9_H_4_N_2_O_4_)(H_2_O)_2_]·H_2_O}_*n*_, chelates to a Sr^II^ atom and at the same time, one of the two O atoms coordinates to a third Sr^II^ atom. The μ_4_-bridging mode of the dianion generates a square-grid layer motif; adjacent layers are connected by O—H⋯O, O—H⋯N and N—H⋯O hydrogen bonds, forming a three-dimensional network. The eight-coordinate Sr atom exists in a distorted square-anti­prismatic geometry. The crystal studied was a non-merohedral twin with a minor twin component of 24%.

## Related literature

For the crystal structures of other metal salts of dicarboxylic acid, see: Gao *et al.* (2008 ▶); Lo *et al.* (2007 ▶); Song *et al.* (2009*a* ▶,*b* ▶). For the treated of diffraction data of twinned crystals, see: Spek (2009 ▶).
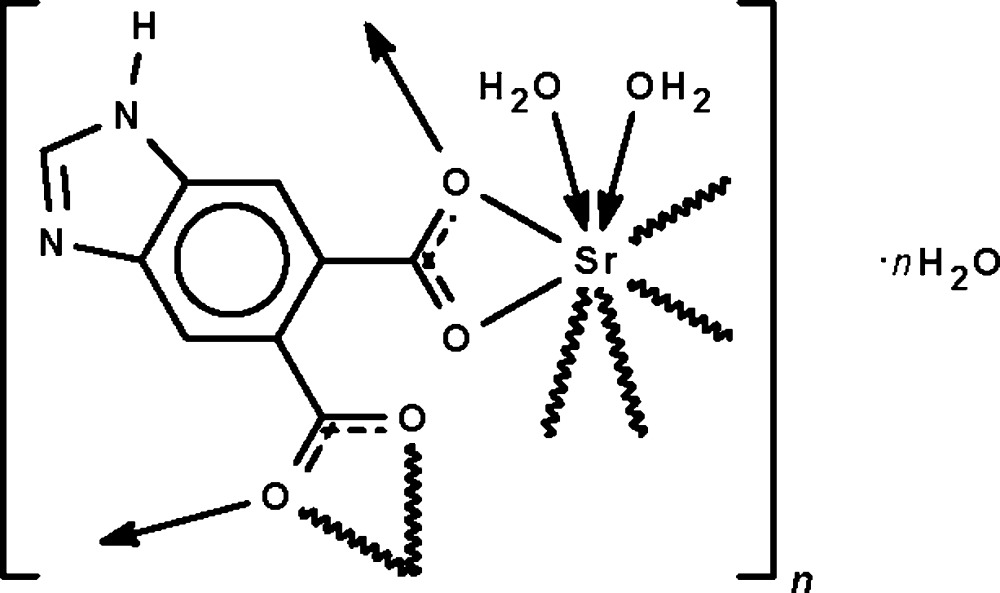


## Experimental

### 

#### Crystal data

[Sr(C_9_H_4_N_2_O_4_)(H_2_O)_2_]·H_2_O
*M* *_r_* = 345.81
Triclinic, 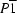

*a* = 6.909 (1) Å
*b* = 7.093 (1) Å
*c* = 13.037 (2) Å
α = 80.860 (5)°
β = 83.974 (5)°
γ = 71.795 (4)°
*V* = 598.2 (2) Å^3^
*Z* = 2
Mo *K*α radiation
μ = 4.54 mm^−1^
*T* = 293 K
0.31 × 0.24 × 0.20 mm

#### Data collection

Rigaku R-AXIS RAPID IP diffractometer
Absorption correction: multi-scan (*ABSCOR*; Higashi, 1995 ▶) *T*_min_ = 0.334, *T*_max_ = 0.464
5799 measured reflections
2695 independent reflections
2339 reflections with *I* > 2σ(*I*)
*R*_int_ = 0.075

#### Refinement

*R*[*F* ^2^ > 2σ(*F* ^2^)] = 0.076
*wR*(*F* ^2^) = 0.214
*S* = 1.05
2695 reflections
173 parameters
H-atom parameters constrained
Δρ_max_ = 2.69 e Å^−3^
Δρ_min_ = −2.12 e Å^−3^

### 

Data collection: *RAPID-AUTO* (Rigaku, 1998 ▶); cell refinement: *RAPID-AUTO*; data reduction: *CrystalClear* (Rigaku/MSC, 2002 ▶); program(s) used to solve structure: *SHELXS97* (Sheldrick, 2008 ▶); program(s) used to refine structure: *SHELXL97* (Sheldrick, 2008 ▶); molecular graphics: *OLEX* (Dolomanov *et al.*, 2003 ▶) and *X-SEED* (Barbour, 2001 ▶); software used to prepare material for publication: *publCIF* (Westrip, 2009 ▶).

## Supplementary Material

Crystal structure: contains datablocks global, I. DOI: 10.1107/S1600536809048284/xu2672sup1.cif

Structure factors: contains datablocks I. DOI: 10.1107/S1600536809048284/xu2672Isup2.hkl

Additional supplementary materials:  crystallographic information; 3D view; checkCIF report

## Figures and Tables

**Table 1:** Selected bond lengths (Å)

Sr1—O1	2.604 (5)
Sr1—O2	2.760 (5)
Sr1—O2^i^	2.516 (5)
Sr1—O3^ii^	2.711 (5)
Sr1—O3^iii^	2.528 (5)
Sr1—O4^ii^	2.635 (6)
Sr1—O1*w*	2.554 (5)
Sr1—O2*w*	2.579 (6)

Symmetry codes: (i) 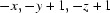; (ii) 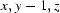; (iii) 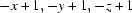.

**Table 2:** Hydrogen-bond geometry (Å, °)

*D*—H⋯*A*	*D*—H	H⋯*A*	*D*⋯*A*	*D*—H⋯*A*
O1*w*—H11⋯O1^iii^	0.84	1.98	2.81 (1)	167
O1*w*—H12⋯O4^iv^	0.84	2.00	2.83 (1)	173
O2*w*—H21⋯O3*w* ^v^	0.84	2.28	2.95 (1)	136
O2*w*—H22⋯O4^i^	0.84	2.12	2.93 (1)	162
O3*w*—H3*w*1⋯N1	0.84	1.97	2.78 (1)	160
O3*w*—H3*w*2⋯O3*w* ^vi^	0.84	2.39	3.01 (2)	132
N2—H2n⋯O3*w* ^vii^	0.88	2.07	2.75 (1)	134

Symmetry codes: (i) 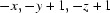; (iii) 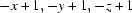; (iv) 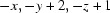; (v) 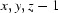; (vi) 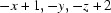; (vii) 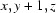.

**Fig. 1.**
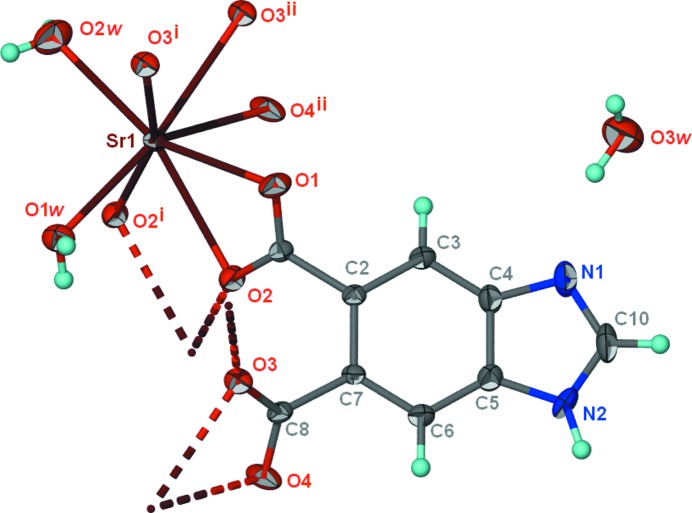
Thermal ellipsoid plot (Barbour, 2001) of a portion of the chain structure of Sr(H2O)2(C9H4N2O2).H2O at the 50% probability level; hydrogen atoms are drawn as spheres of arbitrary radius.

**Fig. 2.**
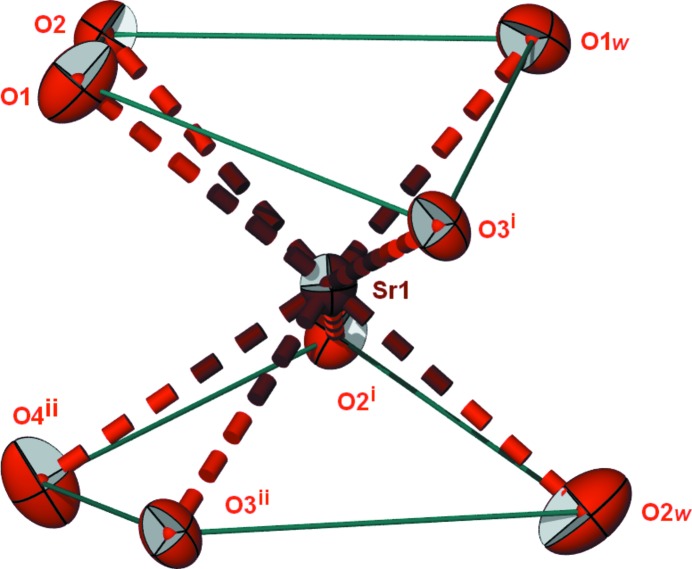
Detail of the geometry of Sr1.

**Fig. 3.**
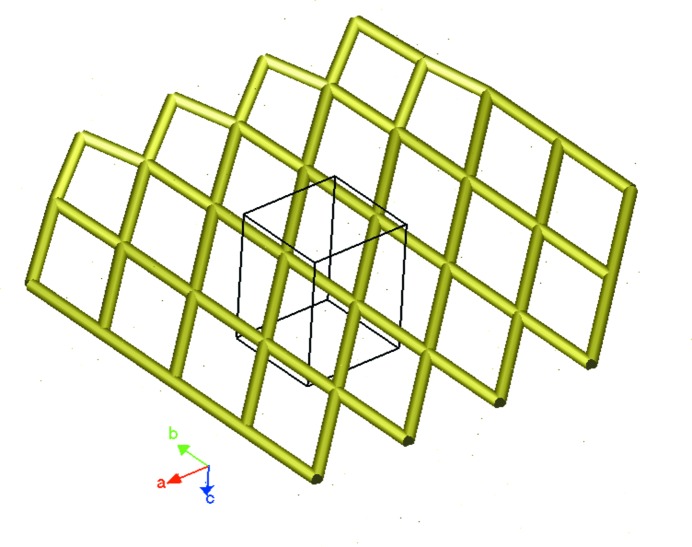
OLEX (Dolomanov et al., 2003) depiction of the layer motif.

## supplementary crystallographic information

### Experimental

Strontium dichloride hexahydrate (0.027 g, 0.1 mmol), 1*H*-benzimidazole-5,6-dicarboxylic acid (0.021 g, 0.1 mmol) and water (15 ml) along with a few drops of sodium hydroxide solution that adjusted the pH to about 7 were placed in a 25 ml glass vessel, which was kept at 277 K for several weeks. Colorless block-shaped crystals were obtained in 60% yield.

### Refinement

Carbon- and nitrogen bound H-atoms were generated geometrically, and were constrained to ride on their parent atoms (C–H = 0.93 Å, *U* = 1.5*U*_eq_(C); N–H 0.88 Å, *U* = 1.2*U*_eq_(N)).

For the two coordinated water molecules, their H-atoms rotated to fit the electron density. For the free water molecule, their H-atoms were placed in chemically sensible positions on the basis of hydrogen bonding interactions; O–H = 0.84 Å. Their temperature factors were similarly tied. The short intermolecular H3*w*2···H3*w*2 contact of < 2.0 Å may be an artifact of possible disorder in the O3*w* water molecule. However, it was not necessary, to split it into two components.

The structure is a non-merohedral twin; the diffraction intensities were split into two components by *PLATON* (Spek, 2009).

The final difference Fourier map had a large peak/deep hole in the vicinity of Sr1.

### Crystal data

**Table:**

[Sr(C_9_H_4_N_2_O_4_)(H_2_O)_2_]·H_2_O	*Z* = 2
*M**_r_* = 345.81	*F*(000) = 344
Triclinic, *P*1	*D*_x_ = 1.920 Mg m^−^^3^
Hall symbol: -P 1	Mo *K*α radiation, λ = 0.71073 Å
*a* = 6.909 (1) Å	Cell parameters from 5129 reflections
*b* = 7.093 (1) Å	θ = 3.1–27.5°
*c* = 13.037 (2) Å	µ = 4.54 mm^−^^1^
α = 80.860 (5)°	*T* = 293 K
β = 83.974 (5)°	Block, colorless
γ = 71.795 (4)°	0.31 × 0.24 × 0.20 mm
*V* = 598.2 (2) Å^3^	

### Data collection

**Table:**

Rigaku R-AXIS RAPID IP diffractometer	2695 independent reflections
Radiation source: fine-focus sealed tube	2339 reflections with *I* > 2σ(*I*)
graphite	*R*_int_ = 0.075
ω scan	θ_max_ = 27.5°, θ_min_ = 3.1°
Absorption correction: multi-scan (*ABSCOR*; Higashi, 1995)	*h* = −8→8
*T*_min_ = 0.334, *T*_max_ = 0.464	*k* = −9→9
5799 measured reflections	*l* = −16→16

### Refinement

**Table:**

Refinement on *F*^2^	Primary atom site location: structure-invariant direct methods
Least-squares matrix: full	Secondary atom site location: difference Fourier map
*R*[*F*^2^ > 2σ(*F*^2^)] = 0.076	Hydrogen site location: inferred from neighbouring sites
*wR*(*F*^2^) = 0.214	H-atom parameters constrained
*S* = 1.05	*w* = 1/[σ^2^(*F*_o_^2^) + (0.1352*P*)^2^ + 1.3937*P*] where *P* = (*F*_o_^2^ + 2*F*_c_^2^)/3
2695 reflections	(Δ/σ)_max_ = 0.001
173 parameters	Δρ_max_ = 2.69 e Å^−^^3^
0 restraints	Δρ_min_ = −2.12 e Å^−^^3^

### Fractional atomic coordinates and isotropic or equivalent isotropic displacement parameters (Å^2^)

**Table:**

	*x*	*y*	*z*	*U*_iso_*/*U*_eq_	
Sr1	0.25457 (8)	0.25734 (8)	0.46107 (4)	0.0207 (3)	
O1	0.4011 (8)	0.3453 (8)	0.6171 (5)	0.0353 (13)	
O2	0.1154 (7)	0.5758 (7)	0.5744 (4)	0.0261 (10)	
O3	0.3642 (7)	0.8869 (7)	0.5721 (4)	0.0268 (10)	
O4	0.0729 (8)	1.0928 (8)	0.6225 (5)	0.0401 (14)	
O1w	0.2542 (8)	0.5682 (8)	0.3316 (4)	0.0327 (12)	
H11	0.3596	0.6002	0.3370	0.049*	
H12	0.1502	0.6631	0.3443	0.049*	
O2w	0.2942 (10)	0.0310 (10)	0.3189 (6)	0.0502 (17)	
H21	0.3087	0.0953	0.2602	0.075*	
H22	0.1896	−0.0064	0.3213	0.075*	
O3w	0.3469 (12)	0.0350 (11)	1.0913 (6)	0.0594 (19)	
H3w1	0.3165	0.1485	1.0555	0.089*	
H3w2	0.4687	−0.0248	1.0740	0.089*	
N1	0.2543 (11)	0.4413 (11)	1.0195 (5)	0.0378 (16)	
N2	0.1892 (12)	0.7679 (11)	1.0241 (5)	0.0387 (16)	
H2N	0.1640	0.8830	1.0480	0.046*	
C1	0.2549 (10)	0.4986 (9)	0.6338 (5)	0.0198 (12)	
C2	0.2500 (9)	0.5836 (9)	0.7336 (5)	0.0192 (12)	
C3	0.2665 (11)	0.4524 (10)	0.8249 (6)	0.0254 (14)	
H3	0.2916	0.3160	0.8239	0.031*	
C4	0.2448 (11)	0.5298 (11)	0.9184 (6)	0.0281 (15)	
C5	0.2019 (11)	0.7366 (11)	0.9214 (6)	0.0276 (14)	
C6	0.1836 (11)	0.8687 (10)	0.8295 (6)	0.0267 (14)	
H6	0.1550	1.0055	0.8308	0.032*	
C7	0.2086 (9)	0.7926 (9)	0.7361 (5)	0.0190 (12)	
C8	0.2165 (9)	0.9307 (9)	0.6363 (5)	0.0216 (13)	
C10	0.2225 (14)	0.5909 (14)	1.0781 (6)	0.042 (2)	
H10	0.2243	0.5692	1.1503	0.051*	

### Atomic displacement parameters (Å^2^)

**Table:**

	*U*^11^	*U*^22^	*U*^33^	*U*^12^	*U*^13^	*U*^23^
Sr1	0.0175 (4)	0.0190 (4)	0.0219 (4)	−0.0002 (2)	−0.0012 (2)	−0.0025 (3)
O1	0.027 (3)	0.032 (3)	0.042 (3)	0.008 (2)	−0.010 (2)	−0.017 (2)
O2	0.022 (2)	0.028 (2)	0.026 (3)	−0.0030 (18)	−0.0043 (19)	−0.007 (2)
O3	0.020 (2)	0.026 (2)	0.029 (3)	−0.0029 (18)	0.0065 (19)	−0.002 (2)
O4	0.030 (3)	0.026 (3)	0.045 (3)	0.011 (2)	0.010 (2)	0.005 (2)
O1w	0.026 (3)	0.030 (3)	0.039 (3)	−0.006 (2)	0.004 (2)	−0.004 (2)
O2w	0.047 (4)	0.051 (4)	0.057 (4)	−0.009 (3)	−0.012 (3)	−0.024 (3)
O3w	0.066 (5)	0.045 (4)	0.066 (5)	−0.016 (3)	−0.013 (4)	0.001 (4)
N1	0.049 (4)	0.045 (4)	0.020 (3)	−0.018 (3)	−0.005 (3)	0.000 (3)
N2	0.055 (4)	0.039 (4)	0.024 (3)	−0.011 (3)	0.000 (3)	−0.017 (3)
C1	0.018 (3)	0.020 (3)	0.022 (3)	−0.004 (2)	0.000 (2)	−0.005 (2)
C2	0.014 (3)	0.019 (3)	0.022 (3)	−0.001 (2)	−0.002 (2)	−0.005 (2)
C3	0.032 (4)	0.020 (3)	0.027 (4)	−0.009 (2)	−0.002 (3)	−0.005 (3)
C4	0.035 (4)	0.030 (4)	0.021 (4)	−0.012 (3)	−0.005 (3)	−0.001 (3)
C5	0.033 (4)	0.027 (3)	0.025 (4)	−0.011 (3)	−0.003 (3)	−0.005 (3)
C6	0.034 (4)	0.019 (3)	0.026 (4)	−0.007 (3)	0.000 (3)	−0.004 (3)
C7	0.020 (3)	0.015 (3)	0.021 (3)	−0.004 (2)	0.002 (2)	−0.003 (2)
C8	0.016 (3)	0.021 (3)	0.025 (3)	0.000 (2)	−0.001 (2)	−0.008 (3)
C10	0.049 (5)	0.058 (5)	0.018 (4)	−0.013 (4)	−0.006 (3)	0.000 (3)

### Geometric parameters (Å, °)

**Table:**

Sr1—O1	2.604 (5)	O2w—H22	0.8400
Sr1—O2	2.760 (5)	O3w—H3w1	0.8400
Sr1—O2^i^	2.516 (5)	O3w—H3w2	0.8400
Sr1—O3^ii^	2.711 (5)	N1—C10	1.354 (11)
Sr1—O3^iii^	2.528 (5)	N1—C4	1.366 (9)
Sr1—O4^ii^	2.635 (6)	N2—C10	1.302 (11)
Sr1—O1w	2.554 (5)	N2—C5	1.381 (9)
Sr1—O2w	2.579 (6)	N2—H2N	0.8800
O1—C1	1.262 (8)	C1—C2	1.511 (9)
O2—C1	1.233 (8)	C2—C3	1.382 (10)
O2—Sr1^i^	2.516 (5)	C2—C7	1.424 (8)
O3—C8	1.242 (8)	C3—C4	1.390 (10)
O3—Sr1^iii^	2.528 (5)	C3—H3	0.9300
O3—Sr1^iv^	2.711 (5)	C4—C5	1.409 (9)
O4—C8	1.264 (8)	C5—C6	1.390 (10)
O4—Sr1^iv^	2.635 (6)	C6—C7	1.383 (9)
O1w—H11	0.8400	C6—H6	0.9300
O1w—H12	0.8400	C7—C8	1.506 (9)
O2w—H21	0.8400	C10—H10	0.9300
			
O2^i^—Sr1—O3^iii^	159.81 (17)	Sr1—O1w—H12	109.5
O2^i^—Sr1—O1w	75.51 (17)	H11—O1w—H12	109.5
O3^iii^—Sr1—O1w	90.53 (16)	Sr1—O2w—H21	109.5
O2^i^—Sr1—O2w	90.93 (18)	Sr1—O2w—H22	109.5
O3^iii^—Sr1—O2w	75.39 (19)	H21—O2w—H22	109.5
O1w—Sr1—O2w	94.2 (2)	H3w1—O3w—H3w2	107.1
O2^i^—Sr1—O1	118.96 (15)	C10—N1—C4	106.1 (7)
O3^iii^—Sr1—O1	77.00 (17)	C10—N2—C5	105.4 (7)
O1w—Sr1—O1	98.63 (19)	C10—N2—H2N	127.3
O2w—Sr1—O1	149.54 (18)	C5—N2—H2N	127.3
O2^i^—Sr1—O4^ii^	78.71 (17)	O2—C1—O1	122.9 (6)
O3^iii^—Sr1—O4^ii^	118.75 (16)	O2—C1—C2	119.6 (6)
O1w—Sr1—O4^ii^	148.09 (16)	O1—C1—C2	117.4 (6)
O2w—Sr1—O4^ii^	104.7 (2)	C3—C2—C7	120.5 (6)
O1—Sr1—O4^ii^	77.8 (2)	C3—C2—C1	117.0 (5)
O2^i^—Sr1—O3^ii^	118.90 (16)	C7—C2—C1	122.2 (6)
O3^iii^—Sr1—O3^ii^	73.35 (17)	C2—C3—C4	118.1 (6)
O1w—Sr1—O3^ii^	163.41 (16)	C2—C3—H3	120.9
O2w—Sr1—O3^ii^	78.2 (2)	C4—C3—H3	120.9
O1—Sr1—O3^ii^	81.86 (17)	N1—C4—C3	132.1 (7)
O4^ii^—Sr1—O3^ii^	48.37 (14)	N1—C4—C5	106.2 (6)
O2^i^—Sr1—O2	72.84 (17)	C3—C4—C5	121.7 (7)
O3^iii^—Sr1—O2	117.87 (16)	N2—C5—C6	131.4 (7)
O1w—Sr1—O2	74.33 (16)	N2—C5—C4	108.4 (6)
O2w—Sr1—O2	161.91 (19)	C6—C5—C4	120.1 (7)
O1—Sr1—O2	48.13 (14)	C7—C6—C5	118.6 (6)
O4^ii^—Sr1—O2	80.35 (17)	C7—C6—H6	120.7
O3^ii^—Sr1—O2	116.38 (15)	C5—C6—H6	120.7
O1—Sr1—C8^ii^	76.73 (18)	C6—C7—C2	121.0 (6)
C1—O1—Sr1	97.5 (4)	C6—C7—C8	118.7 (5)
C1—O2—Sr1^i^	152.2 (5)	C2—C7—C8	119.9 (6)
C1—O2—Sr1	90.8 (4)	O3—C8—O4	122.0 (7)
Sr1^i^—O2—Sr1	107.16 (17)	O3—C8—C7	120.0 (5)
C8—O3—Sr1^iii^	148.1 (5)	O4—C8—C7	117.9 (6)
C8—O3—Sr1^iv^	92.8 (4)	N2—C10—N1	113.9 (7)
Sr1^iii^—O3—Sr1^iv^	106.65 (17)	N2—C10—H10	123.0
C8—O4—Sr1^iv^	95.9 (4)	N1—C10—H10	123.0
Sr1—O1w—H11	109.5		
			
O2^i^—Sr1—O1—C1	−13.7 (5)	C1—C2—C3—C4	−174.6 (6)
O3^iii^—Sr1—O1—C1	153.0 (5)	C10—N1—C4—C3	179.6 (8)
O1w—Sr1—O1—C1	64.5 (5)	C10—N1—C4—C5	−1.3 (9)
O2w—Sr1—O1—C1	178.4 (4)	C2—C3—C4—N1	−179.4 (8)
O4^ii^—Sr1—O1—C1	−83.2 (4)	C2—C3—C4—C5	1.6 (10)
O3^ii^—Sr1—O1—C1	−132.3 (5)	C10—N2—C5—C6	−178.7 (9)
O2—Sr1—O1—C1	4.7 (4)	C10—N2—C5—C4	0.0 (9)
O2^i^—Sr1—O2—C1	158.5 (5)	N1—C4—C5—N2	0.8 (9)
O3^iii^—Sr1—O2—C1	−40.1 (4)	C3—C4—C5—N2	−180.0 (7)
O1w—Sr1—O2—C1	−122.2 (4)	N1—C4—C5—C6	179.7 (7)
O2w—Sr1—O2—C1	−174.5 (6)	C3—C4—C5—C6	−1.1 (11)
O1—Sr1—O2—C1	−4.8 (4)	N2—C5—C6—C7	178.5 (8)
O4^ii^—Sr1—O2—C1	77.4 (4)	C4—C5—C6—C7	−0.1 (11)
O3^ii^—Sr1—O2—C1	44.2 (4)	C5—C6—C7—C2	0.7 (10)
O2^i^—Sr1—O2—Sr1^i^	0.0	C5—C6—C7—C8	−172.0 (6)
O3^iii^—Sr1—O2—Sr1^i^	161.42 (17)	C3—C2—C7—C6	−0.2 (9)
O1w—Sr1—O2—Sr1^i^	79.3 (2)	C1—C2—C7—C6	173.1 (6)
O2w—Sr1—O2—Sr1^i^	27.0 (7)	C3—C2—C7—C8	172.4 (6)
O1—Sr1—O2—Sr1^i^	−163.2 (3)	C1—C2—C7—C8	−14.3 (9)
O4^ii^—Sr1—O2—Sr1^i^	−81.1 (2)	Sr1^iii^—O3—C8—O4	138.1 (7)
O3^ii^—Sr1—O2—Sr1^i^	−114.29 (18)	Sr1^iv^—O3—C8—O4	9.6 (7)
Sr1^i^—O2—C1—O1	139.9 (8)	Sr1^iii^—O3—C8—C7	−39.6 (12)
Sr1—O2—C1—O1	8.8 (7)	Sr1^iv^—O3—C8—C7	−168.1 (5)
Sr1^i^—O2—C1—C2	−37.6 (12)	Sr1^iv^—O4—C8—O3	−9.9 (7)
Sr1—O2—C1—C2	−168.8 (5)	Sr1^iv^—O4—C8—C7	167.8 (5)
Sr1—O1—C1—O2	−9.4 (8)	C6—C7—C8—O3	125.0 (7)
Sr1—O1—C1—C2	168.3 (5)	C2—C7—C8—O3	−47.8 (9)
O2—C1—C2—C3	125.8 (7)	C6—C7—C8—O4	−52.8 (9)
O1—C1—C2—C3	−51.9 (9)	C2—C7—C8—O4	134.4 (7)
O2—C1—C2—C7	−47.7 (9)	C5—N2—C10—N1	−0.9 (10)
O1—C1—C2—C7	134.6 (7)	C4—N1—C10—N2	1.4 (10)
C7—C2—C3—C4	−0.9 (9)		

Symmetry codes: (i) −*x*, −*y*+1, −*z*+1; (ii) *x*, *y*−1, *z*; (iii) −*x*+1, −*y*+1, −*z*+1; (iv) *x*, *y*+1, *z*.

### Hydrogen-bond geometry (Å, °)

**Table:**

*D*—H···*A*	*D*—H	H···*A*	*D*···*A*	*D*—H···*A*
O1w—H11···O1^iii^	0.84	1.98	2.81 (1)	167
O1w—H12···O4^v^	0.84	2.00	2.83 (1)	173
O2w—H21···O3w^vi^	0.84	2.28	2.95 (1)	136
O2w—H22···O4^i^	0.84	2.12	2.93 (1)	162
O3w—H3w1···N1	0.84	1.97	2.78 (1)	160
O3w—H3w2···O3w^vii^	0.84	2.39	3.01 (2)	132
N2—H2n···O3w^iv^	0.88	2.07	2.75 (1)	134

Symmetry codes: (iii) −*x*+1, −*y*+1, −*z*+1; (v) −*x*, −*y*+2, −*z*+1; (vi) *x*, *y*, *z*−1; (i) −*x*, −*y*+1, −*z*+1; (vii) −*x*+1, −*y*, −*z*+2; (iv) *x*, *y*+1, *z*.
